# Optimizing estradiol duration to reduce progesterone-related cancellation in hormone replacement therapy frozen embryo transfer cycles: toward a patient-tailored approach

**DOI:** 10.3389/fendo.2026.1898027

**Published:** 2026-07-22

**Authors:** Mehmet Resit Asoglu, Ezgi Darıcı Kurt, Gurkan Bozdag, Kubra Boynukalin, Ozkan Ozdamar, Mustafa Bahceci

**Affiliations:** 1 Bahceci IVF center, Istanbul, Türkiye; 2 Department of Obstetrics and Gynecology, Uskudar University, Istanbul, Türkiye

**Keywords:** cycle cancellation, frozen-thawed embryo transfer, hormone replacement therapy, live birth rate, progesterone elevation

## Abstract

**Objective:**

To study whether the duration of estradiol exposure in hormone replacement therapy (HRT) cycles for frozen embryo transfer (FET) is associated with progesterone-related cycle cancellation and reproductive outcomes.

**Materials and methods:**

This retrospective cohort study included 2, 001 HRT–FET cycles following a freeze-all strategy performed between January 2020 and June 2025. Patients were stratified according to estradiol exposure duration into short (8–9 days; n = 980), intermediate (10–11 days; n = 746), and prolonged (12–14 days; n = 275) exposure groups. Serum progesterone levels were assessed on the day of progesterone initiation. The primary outcome was cycle cancellation due to progesterone elevation (progesterone ≥1.5 ng/mL on the day of progesterone initiation). Secondary outcomes included implantation, clinical pregnancy, miscarriage, and live birth rates.

**Results:**

The overall cycle cancellation rate was 3.3% (66/2, 001) and increased significantly with longer estradiol exposure, occurring in 2.1%, 3.9%, and 5.8% of cycles in the short, intermediate, and prolonged groups, respectively (OR 1.69 per category, 95% CI 1.22-2.34, p = 0.002). Live birth rates per transfer were similar across groups (47.2%, 52.3%, and 49.4%, respectively; p = 0.122). In multivariable analysis, prolonged estradiol exposure (OR 3.68, 95% CI 1.76–7.71, p < 0.001), intermediate exposure (OR 2.08, 95% CI 1.13–3.81, p = 0.019), age >37 years (OR 2.58, 95% CI 1.36–4.90, p = 0.004), and diminished ovarian reserve (OR 5.13, 95% CI 2.87–9.16, p < 0.001) were independent predictors of cycle cancellation, whereas polycystic ovary syndrome (OR 0.23, 95% CI 0.08–0.69, p = 0.009) was independently associated with a lower likelihood of cancellation. In the multivariable model restricted to transferred cycles, neither intermediate nor prolonged estradiol exposure was independently associated with live birth compared with short estradiol exposure (intermediate: OR 1.25, 95% CI 0.88–1.78, p = 0.204; prolonged: OR 0.93, 95% CI 0.57–1.51, p = 0.775).

**Conclusions:**

Prolonged estradiol exposure in HRT–FET cycles is associated with increased progesterone-related cycle cancellation without improving reproductive outcomes. A patient-tailored approach based on ovarian reserve status and age may help reduce avoidable cancellations.

## Introduction

The clinical utility of freeze-all strategies in assisted reproductive technology has increased substantially with advances in laboratory techniques, particularly vitrification (1). By deferring embryo transfer to a subsequent cycle, this approach minimizes the potential adverse effects of supraphysiologic hormone levels during ovarian stimulation on endometrial receptivity and may improve embryo–endometrium synchrony (2–4). Consequently, frozen–thawed embryo transfer (FET) has demonstrated favorable reproductive outcomes in selected patient populations (3–7).

Hormone replacement therapy (HRT) cycles are commonly used for endometrial preparation in FET, offering flexible scheduling, simplified clinical management, and pregnancy outcomes comparable to ovulatory or stimulated cycles (8). In HRT–FET cycles, endometrial preparation is achieved with exogenous estradiol followed by progesterone to induce secretory transformation (9). Despite these advantages, emerging evidence suggests that HRT–FET cycles may be associated with an increased risk of adverse reproductive and obstetric outcomes, including miscarriage and hypertensive disorders of pregnancy (9–12).

A key challenge in HRT–FET cycles is unintended progesterone elevation before planned progesterone initiation, which often leads to cycle cancellation due to concerns about impaired embryo–endometrium synchrony. Cycle cancellation imposes a substantial clinical burden by increasing patient distress, prolonging time to pregnancy, and raising treatment costs. These issues highlight the need for strategies to reduce avoidable cancellations and re-evaluate current HRT–FET practices.

To date, the relationship between estradiol exposure duration, progesterone-related cycle cancellation, and reproductive outcomes has not been systematically evaluated, and data addressing these outcomes within the same cohort are limited. We hypothesized that prolonged estradiol exposure in HRT–FET cycles increase progesterone-related cancellations without improving pregnancy outcomes and that a patient-tailored approach could reduce cancellations without compromising results. Accordingly, this study assessed the impact of estradiol exposure duration on cycle cancellation and reproductive outcomes in freeze-all HRT–FET cycles across short (8–9 days), intermediate (10–11 days), and prolonged (12–14 days) exposure groups.

## Materials and methods

### Study design and population

This retrospective cohort study was conducted at Bahceci IVF Center (Istanbul, Turkiye) between January 2020 and June 2025. The study included freeze-all cycles scheduled for HRT–FET that resulted in high- or average-quality blastocyst transfer, as well as cycles cancelled due to progesterone elevation during the same study period. The study protocol was approved by the local institutional review board. Clinical and laboratory data were extracted from the center’s electronic medical record system and manually verified to ensure completeness and accuracy.

**Figure 1:**
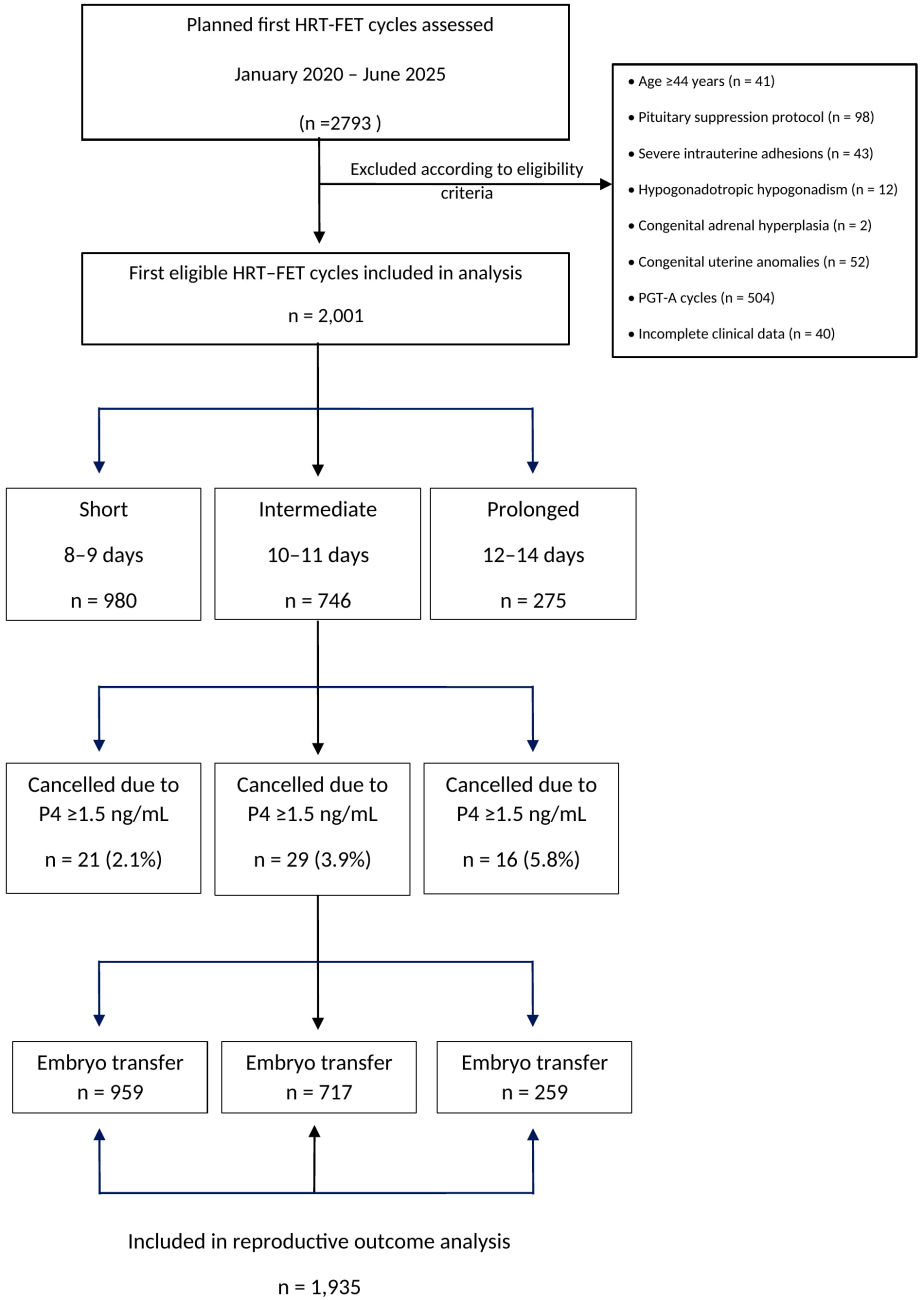
STROBE checklist for the reporting of this retrospective cohort study.

Eligible cases included women younger than 44 years who were scheduled to undergo HRT–FET cycles following a gonadotropin-releasing hormone (GnRH) antagonist stimulation protocol. Only cycles with a normal uterine cavity—documented either before treatment initiation or following corrective uterine surgery—were included. Cycles were excluded if they involved pituitary suppression protocols, were complicated by severe intrauterine adhesions, or met any of the following criteria: hypogonadotropic hypogonadism, congenital adrenal hyperplasia, congenital uterine anomalies (including unicornuate, bicornuate, and didelphys uteri), or incomplete clinical data. Cycles involving preimplantation genetic testing for aneuploidy were also excluded to minimize bias in calculating reproductive outcomes (**Figure 1**).

### Laboratory procedures

Ovarian stimulation was initiated on cycle days 2–3 using gonadotropins. A GnRH antagonist protocol was employed, with daily cetrorelix acetate (0.25 mg) commenced when the leading follicle reached 12–14 mm in diameter. Final oocyte maturation was triggered with 0.2 mg triptorelin acetate, 250 micrograms recombinant hCG, or a combination of hCG and triptorelin acetate when at least two follicles reached a diameter of ≥18 mm. Transvaginal oocyte retrieval was performed 35 or 36 hours after trigger administration.

Retrieved cumulus–oocyte complexes were incubated for 2 hours at 37 °C in an atmosphere of 6% CO_2_ and 5% O_2_, followed by enzymatic and mechanical denudation. Oocytes were subsequently inseminated using Continuous Single Culture Medium (CSCM-Complete^®^, Irvine Scientific, CA, USA).

Blastocyst development and morphological quality were evaluated using the Gardner and Schoolcraft classification system (13). Blastocysts graded 3–6 with AA, AB, BA, or BB morphology were classified as high quality, whereas those graded 3–6 BC were considered average quality. Embryo quality assessment was performed with identical grading criteria applied to both day 5 and day 6 blastocysts. Cryopreservation and warming were carried out using commercially available vitrification and thawing kits (Vit Kit^®^-Freeze and Vit Kit^®^-Thaw, Irvine Scientific, CA, USA) in accordance with the manufacturer’s instructions.

### Endometrial preparation and HRT–FET protocols

Baseline transvaginal ultrasonography and serum hormone assessment were performed on cycle day 2 or 3 to confirm low endogenous hormonal activity consistent with early follicular phase levels, defined as estradiol (E2) concentrations <80 pg/mL, progesterone (P4) concentrations <1.5 ng/mL, and a thin endometrium (<4 mm). Endometrial preparation was achieved using oral estradiol (Estrofem^®^, Novo Nordisk) administered according to one of two HRT regimens. An incremental-dose protocol was employed from January 2020 through October 2023, after which a fixed-dose regimen was implemented. In the incremental-dose protocol, estradiol was initiated at 4 mg daily and increased by 2 mg at four-day intervals to a maximum daily dose of 8 mg. In the fixed-dose regimen, estradiol was administered orally at 2 mg three times daily.

Follow-up transvaginal ultrasonography and repeat serum E2 and P4 measurements were performed after 8 to 14 days of estradiol exposure to assess endometrial adequacy for embryo transfer. The duration of estradiol administration was not predefined and varied according to clinician preference and patient-specific logistical considerations, including occupational commitments, travel requirements, and residence outside the local region.

Progesterone supplementation was initiated once a trilaminar endometrial pattern with a thickness ≥7 mm was observed and serum progesterone levels remained below 1.5 ng/mL. Luteal phase support consisted of subcutaneous progesterone (25 mg twice daily; Prolutex^®^, IBSA). Frozen–thawed blastocyst transfer was performed on the sixth day following progesterone initiation in all cases. In cycles resulting in clinical pregnancy, progesterone supplementation was continued until 10 weeks of gestation.

### Hormone assays

Serum E2 and P4 concentrations were measured in pg/mL and ng/mL, respectively, using automated electrochemiluminescence immunoassays (Elecsys Estradiol and Progesterone III assays; Roche, Canada) on Elecsys and Cobas E analyzers. The lower limits of detection were 5 pg/mL for E2 and 0.05 ng/mL for P4. Intra-assay and inter-assay coefficients of variation were approximately 2.0–3.5% and 2.5–6.5% for E2, and 1.3–2.5% and 2.0–4.8% for P4, respectively.

### Outcome measures

Patients were stratified into three groups based on the duration of estradiol exposure: short (8–9 days), intermediate (10–11 days), and prolonged (12–14 days). Baseline characteristics, including ovarian reserve status, and outcome measures were compared among the three groups. For risk stratification analysis, patients were also categorized by ovarian reserve status. Ovarian reserve status was classified as follows: Diminished ovarian reserve (DOR) was defined as antral follicle count (AFC) <7 follicles or anti-Müllerian hormone (AMH) <1.1 ng/mL (14). Polycystic ovary syndrome (PCOS) was diagnosed according to the Rotterdam criteria (15), requiring at least two of the following: oligo-ovulation or anovulation, clinical or biochemical hyperandrogenism, and polycystic ovarian morphology on ultrasound. Normal ovarian reserve was defined as AFC ≥7 and AMH ≥1.1 ng/mL in the absence of PCOS. The primary outcome was cycle cancellation rate, defined as a serum progesterone level ≥1.5 ng/mL on the day of planned progesterone initiation. Secondary outcomes included implantation, clinical pregnancy, miscarriage, and live birth rates among cycles that proceeded to transfer. Pregnancy was confirmed by a serum β–human chorionic gonadotropin level ≥5 IU/L measured 12 days after embryo transfer. The implantation rate was defined as the proportion of embryo transfer cycles resulting in at least one gestational sac. Biochemical pregnancy was defined as a positive serum β-hCG test that did not progress to ultrasound confirmation of an intrauterine gestational sac. Clinical pregnancy was defined as the presence of an intrauterine fetal heartbeat, and miscarriage was defined as pregnancy loss before 20 weeks of gestation. Miscarriage rate was calculated as the number of miscarriages divided by the number of clinical pregnancies. Live birth was defined as delivery beyond 24 weeks of gestation.

### Statistical analysis

Continuous variables are presented as mean ± standard deviation, and categorical variables as number (percentage). Data distribution was assessed using the Kolmogorov–Smirnov test. Continuous variables were compared using one-way ANOVA with Tukey’s *post hoc* test when appropriate. Categorical variables were compared using Pearson’s chi-square or Fisher’s exact test, with Bonferroni correction applied for pairwise comparisons among more than two groups. Linear-by-linear association test was used to assess linear trends in cycle cancellation rates across exposure groups. Multivariable logistic regression analyses evaluated whether estradiol exposure duration independently predicted cycle cancellation and live birth after adjusting for confounders, including ovarian reserve status (DOR, normal, and PCOS) and treatment protocol (incremental vs. fixed). For regression analysis, embryo quality was classified as high if at least one blastocyst met high-quality morphology criteria in double-blastocyst transfers. Receiver operating characteristic (ROC) curve analyses assessed the discriminative ability of estradiol exposure duration for predicting cycle cancellation, using three sequential models: exposure duration alone, exposure duration combined with age, and exposure duration combined with age and ovarian reserve status. Area under curve (AUC) was calculated and optimal cut-offs determined using the Youden index. Statistical analyses were performed using SPSS version 21.0 (IBM Corp., Armonk, NY, USA); p < 0.05 was considered statistically significant.

## Results

A total of 2, 001 HRT–FET cycles planned for frozen–thawed blastocyst transfer were analyzed: 980 (49.0%) in the short (8–9 days), 746 (37.3%) in the intermediate (10–11 days), and 275 (13.7%) in the prolonged (12–14 days) estradiol exposure groups. To minimize selection bias and avoid within-patient correlation, only the first HRT–FET cycle per patient was included, and all subsequent cycles were excluded from the study. Patients whose first cycle was canceled due to progesterone elevation did not contribute subsequent cycles to pregnancy outcome analyses.

Baseline characteristics are summarized in **Table 1**. Across all patients, mean age was 29.9 ± 4.7 years, BMI 26.4 ± 5.8 kg/m², infertility duration 4.3 ± 2.9 years, and prior IVF attempts with other clinics were 2.2 ± 1.5. Most patients were 37 years or younger (92.9%, n = 1, 859), and 93.8% had primary infertility. Infertility etiologies included unexplained (19.7%), tubal factor (4.9%), PCOS (25.9%), endometrioma (5.6%), DOR (10.4%), male factor (17.4%), and combined factors (16.2%). By ovarian reserve status, 13% (n = 260) had DOR, 47.8% (n = 956) had normal reserve, and 39.2% (n = 785) had PCOS. Baseline characteristics did not differ among the three groups (all p > 0.05).

**Table 1:** Baseline characteristics according to estradiol exposure duration.

	Short (8-9 days) exposure (n=980)	Intermediate (10-11 days) exposure (n=746)	Prolonged (12-14 days) exposure (n=275)	p
Age (years)	29.9 ± 4.6	29.7 ± 4.7	30.2 ± 4.6	0.349
Body mass index (kg/m2)	26.5 ± 5.9	26.2 ± 5.7	26.9 ± 6.1	0.137
Duration of infertility (years)	4.3 ± 2.9	4.2 ± 2.8	4.1 ± 2.9	0.319
Previous IVF attempts	2.2 ± 1.4	2.2 ± 1.4	2.1 ± 1.5	0.673
Primary infertility	916 (93.5)	702 (94.1)	258 (93.8)	0.864
Infertility causes				0.083
Unexplained	193 (19.7)	142 (19.0)	58 (21.1)	
Tubal factor	45 (4.6)	39 (5.2)	15 (5.5)	
Polycystic ovary syndrome	262 (26.7)	190 (25.5)	69 (25.1)	
Endometrioma	40 (4.1)	52 (7.0)	20 (7.3)	
Diminished ovarian reserve	111 (11.3)	66 (8.8)	24 (8.7)	
Male factor infertility	179 (18.3)	118 (15.8)	53 (19.3)	
Combination	150 (15.3)	139 (18.6)	36 (13.1)	
Ovarian reserve status				0.577
Diminished ovarian reserve	133 (13.6)	96 (12.9)	31 (11.3)	
Normal ovarian reserve	468 (47.8)	346 (46.4)	142 (51.6)	
Polycystic ovary syndrome	379 (38.7)	304 (40.8)	102 (37.1)	

Values are given as mean (± standard deviation) or number (percentage).

Cycle- and HRT-related parameters are shown in **Table 2**. No significant differences were observed in stimulation duration, number of oocytes retrieved, metaphase II oocytes, fertilized oocytes, cryopreserved embryos, or trigger-day E2 and P4 levels and endometrial thickness among the three groups (all p > 0.05). In contrast, E2 levels on the day of progesterone initiation differed significantly (p < 0.001), being lower in the short group (370 ± 158 pg/mL) than in the intermediate (434 ± 201 pg/mL) and prolonged (400 ± 171 pg/mL) groups; all pairwise comparisons were statistically significant (**Table 2**). P4 levels also differed among groups (p = 0.001), with lower values in the short versus intermediate group (p < 0.001), while the intermediate–prolonged comparison approached statistical significance (p = 0.066). Endometrial thickness was similar across groups (p = 0.868). Single versus double blastocyst transfer rates did not differ among the groups (p = 0.460). The groups were also similar in terms of the mean number of high-quality and average-quality blastocysts transferred (p = 0.492 and p = 0.822, respectively) (**Table 2**).

**Table 2:** Cycle and HRT–FET parameters according to estradiol exposure duration.

	Short (8-9 days) exposure	Intermediate (10-11 days) exposure	Prolonged (12-14 days) exposure	p
All cycles	(n=980)	(n=746)	(n=275)	
Cycle-related parameters				
Stimulation duration (days)	10.6 ± 1.2	10.7 ± 1.2	10.6 ± 1.2	0.651
Number of oocytes retrieved	17.8 ± 9.4	17.1 ± 9.3	17.6 ± 9.9	0.290
Number of metaphase-2 oocytes	13.8 ± 7.7	13.6 ± 7.8	13.9 ± 8.5	0.725
Number of 2PN oocytes	10.9 ± 6.3	10.8 ± 6.3	11.2 ± 6.4	0.547
Number of blastocysts obtained	4.9 ± 3.4	4.7 ± 3.4	5.0 ± 3.7	0.432
E2 level on trigger day (pg/mL)	2855 ± 2248	2901 ± 2221	2958 ± 2244	0.793
P4 level on trigger day (ng/mL)	0.98 ± 0.76	1.01 ± 0.72	0.99 ± 0.83	0.666
EL on trigger day (mm)	10.1 ± 1.8	9.9 ± 1.9	9.9 ± 2.1	0.563
Transfer cycles	(n=959)	(n=717)	(n=259)	
HRT-related parameters				
Estradiol exposure duration (days)	8.7 ± 0.5	10.5 ± 0.5	12.4 ± 0.6	<0.001
On cycle day 2–3				
E2 level (pg/mL)	43 ± 15	43 ± 16	45 ± 15	0.574
P4 level (ng/mL)	0.36 ± 0.23	0.39 ± 0.22	0.39 ± 0.21	0.130
On progesterone initiation				
E2 level (pg/mL)	370 ± 158	434 ± 201	400 ± 171	<0.001
P4 level (ng/mL)	0.27 ± 0.19	0.31 ± 0.23	0.28 ± 0.18	0.001
EL (mm)	9.6 ± 1.3	9.7 ± 1.4	9.7 ± 1.5	0.868
Number of blastocyst transferred				0.460
Single blastocyst transfer	800 (83.4)	585 (81.6)	219 (84.6)	
Double blastocyst transfer	159 (16.6)	132 (18.4)	40 (15.4)	
Quality of blastocysts transferred				
High-quality	1.11 ± 0.31	1.10 ± 0.30	1.08 ± 0.26	0.492
Average-quality	1.16 ± 0.36	1.14 ± 0.35	1.17 ± 0.37	0.822

Values are given as mean (± standard deviation) or number (percentage).

NA, Not applicable.

The overall cycle cancellation rate was 3.3% (66/2, 001) and increased significantly with longer estradiol exposure, occurring in 2.1%, 3.9%, and 5.8% of cycles in the short, intermediate, and prolonged groups, respectively (OR 1.69 per category, 95% CI 1.22-2.34, p = 0.002). Cancellation rates also differed significantly according to ovarian reserve status, reaching 14.2% in women with DOR, compared with 2.6% in those with normal ovarian reserve and 0.5% in women with PCOS (p < 0.001). Among cycles that proceeded to transfer, pregnancy rates were 71.7%, 75.7%, and 71.4% in the short, intermediate, and prolonged groups, respectively (p = 0.150). The implantation (62.0%, 66.4%, and 61.8%; p = 0.151) and clinical pregnancy rates (56.9%, 61.1%, and 56.0%; p = 0.165) were similar across groups. Live birth rates were slightly higher in the intermediate group (52.3%) compared with the short (47.2%) and prolonged (49.4%) groups, but this difference was not statistically significant (p = 0.122). Miscarriage (14.8%, 14.1%, and 12.4%; p = 0.600) and multiple pregnancy rates (2.7%, 4.5%, and 3.9%; p = 0.148) did not differ among groups (**Table 3**).

**Table 3:** Cycle cancellation and pregnancy outcomes per embryo transfer according to estradiol exposure duration.

	Short (8-9 days) exposure	Intermediate (10-11 days) exposure	Prolonged (12-14 days) exposure	p
All cycles	(n=980)	(n=746)	(n=275)	
Cycle cancellation	2.1 (21)	3.9 (29)	5.8 (16)	0.002
Transfer cycles	(n=959)	(n=717)	(n=259)	
Pregnancy	71.7 (688)	75.7 (543)	71.4 (185)	0.150
Biochemical pregnancy	9.7 (93)	9.1 (65)	9.3 (24)	0.905
Implantation	62 (595)	66.4 (476)	61.8 (160)	0.151
Miscarriage	14.8 (142)	14.1 (101)	12.4 (32)	0.600
Clinical pregnancy	56.9 (546)	61.1 (438)	56 (145)	0.165
Live birth	47.2 (453)	52.3 (375)	49.4 (128)	0.122
Multiple pregnancy	2.7 (26)	4.5 (32)	3.9 (10)	0.148

Values are presented as % (n).

In multivariable analysis (**Table 4**), age >37 years (OR 2.58, 95% CI 1.36–4.90, p = 0.004) and diminished ovarian reserve (OR 5.13, 95% CI 2.87–9.16, p < 0.001) were independently associated with an increased likelihood of cycle cancellation. Compared with short estradiol exposure, both prolonged (OR 3.68, 95% CI 1.76–7.71, p < 0.001) and intermediate exposure (OR 2.08, 95% CI 1.13–3.81, p = 0.019) remained significant predictors. Conversely, PCOS was independently associated with a reduced risk of cancellation (OR 0.23, 95% CI 0.08–0.69, p = 0.009). In a separate multivariable model using PCOS as the reference category, DOR conferred a markedly higher cancellation risk (OR 21.8, 95% CI 7.1–66.8, p < 0.001), underscoring the substantial divergence in risk profiles between these phenotypes.

**Table 4:** Multivariable logistic regression analysis of factors associated with cycle cancellation.

	OR	95%CI	p
Age > 37 years	2.58	1.36-4.90	**0.004**
Body mass index	0.96	0.91-1.02	0.195
Infertility duration	0.97	0.89-1.06	0.527
Protocol (incremental)	Ref		
Fixed protocol	1.20	0.68-2.13	0.524
Normal ovarian reserve	Ref		
Diminished ovarian reserve	5.13	2.87-9.16	**<0.001**
Polycystic ovary syndrome	0.23	0.08-0.69	**0.009**
Short estradiol exposure	Ref		
Intermediate estradiol exposure	2.08	1.13-3.81	**0.019**
Prolonged estradiol exposure	3.68	1.76-7.71	**<0.001**

OR, odds ratio; CI, confidence interval.

Ref, Reference.

Reference categories: normal ovarian reserve and short estradiol exposure (8–9 days). Bold values indicate statistically significant associations (p < 0.05).

In the multivariable model restricted to transferred cycles (**Table 5**), neither intermediate nor prolonged estradiol exposure was independently associated with live birth compared with short estradiol exposure (intermediate: OR 1.25, 95% CI 0.88–1.78, p = 0.204; prolonged: OR 0.93, 95% CI 0.57–1.51, p = 0.775). As expected, age ≤37 years, double blastocyst transfer, and the presence of at least one high-quality blastocyst were independently associated with higher live birth rates (p = 0.015, p < 0.001, and p < 0.001, respectively).

**Table 5:** Multivariable logistic regression analysis of factors associated with live birth.

	OR	95%CI	p
Age < 38 years	2.11	1.16-3.83	**0.015**
Body mass index	1.01	0.98-1.04	0.531
Infertility duration	1.00	0.95-1.05	0.888
Previous IVF attempts	0.99	0.88-1.11	0.875
Protocol (incremental)	Ref		
Fixed protocol	0.96	0.67-1.37	0.820
Normal ovarian reserve	Ref		
Diminished ovarian reserve	0.85	0.54-1.33	0.474
Polycystic ovary syndrome	0.93	0.65-1.35	0.717
Double blastocyt transfer	2.29	1.57-3.34	**<0.001**
High-quality blastocyst transfer	1.83	1.29-2.58	**<0.001**
On progesterone initiation			
Endometrial thickness	1.08	0.96-1.21	0.191
Estradiol level	1.00	1.00-1.00	0.710
Progesterone level	1.1	0.51-2.35	0.815
Short estradiol exposure	Ref		
Intermediate estradiol exposure	1.25	0.88-1.78	0.204
Prolonged estradiol exposure	0.93	0.57-1.51	0.775

OR and CI denote odds ratio and confidence interval. Bold values indicate statistically significant associations (p < 0.05).

**Figure 2:**
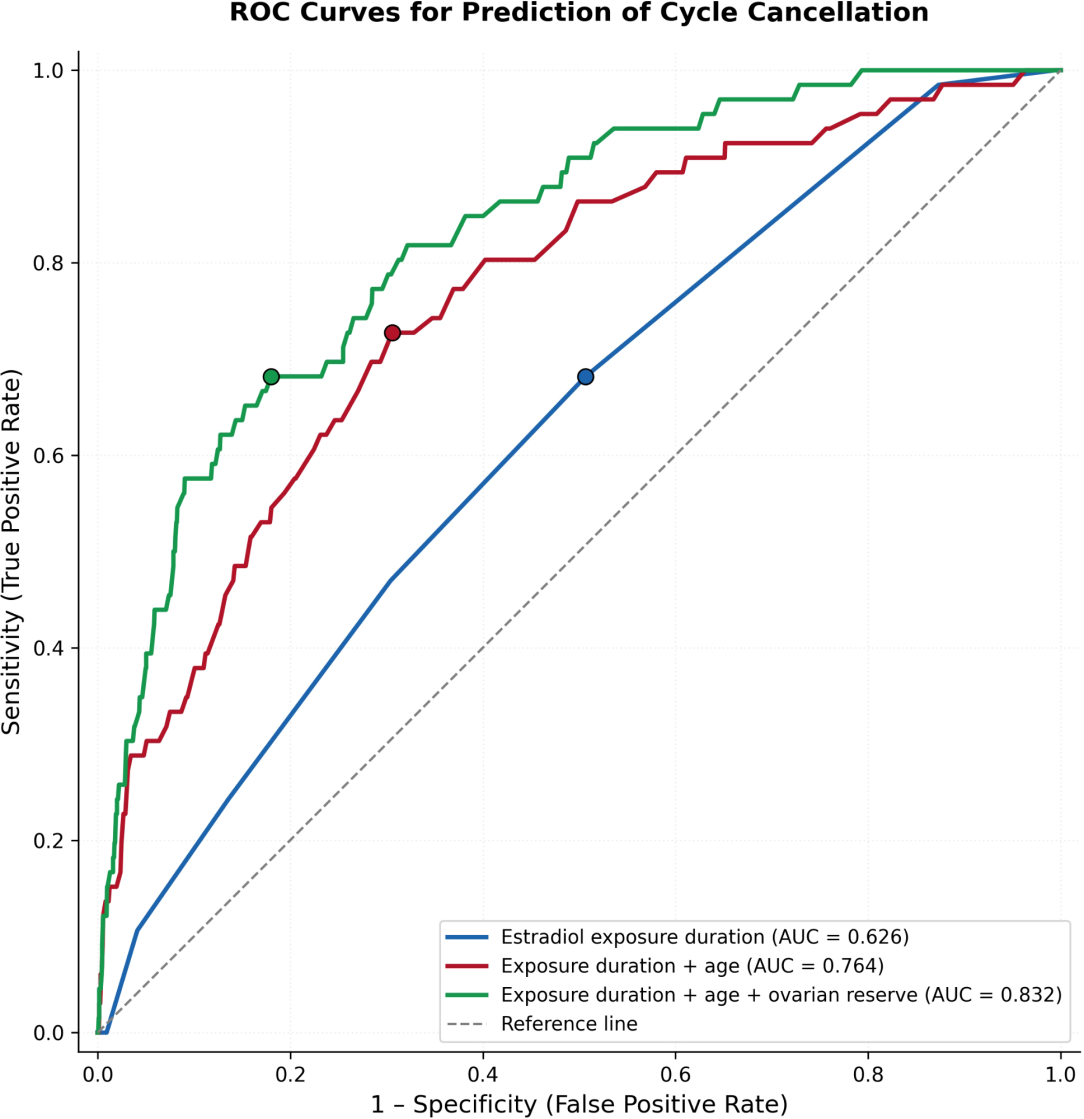
Receiver operating characteristic (ROC) curves for estradiol exposure duration alone, combined with age, and combined with age and ovarian reserve status, for prediction of cycle cancellation.

ROC analysis (**Figure 2**) showed that estradiol exposure duration alone moderately predicted cycle cancellation (AUC 0.626, 95% CI 0.556–0.688, *p* < 0.001), with an optimal cut-off of 10 days (68.2% sensitivity, 49.3% specificity). This 10-day threshold should be considered a finding derived from the present dataset and requires external validation before clinical application. Age was entered into the combined model as a continuous variable, and combining estradiol exposure duration with age improved discrimination (AUC 0.764, 95% CI 0.699–0.817, *p* < 0.001; sensitivity 72.7%, specificity 69.4%). A third model additionally including ovarian reserve as a categorical variable further improved the predictive performance (AUC 0.832, 95% CI 0.783–0.877, *p* < 0.001; sensitivity 68.2%, specificity 82.8%).

In subgroup analyses stratified by ovarian reserve, age, and estradiol exposure duration (**Table 6**), among patients with DOR, cancellation rates were 9.8% (95% CI, 5.3–16.1), 18.8% (95% CI, 11.5–28.0), and 19.4% (95% CI, 7.5–37.5) in the short, intermediate, and prolonged exposure groups, respectively. Among women older than 37 years, the corresponding rates were 7.8% (95% CI, 2.9–16.2), 19.1% (95% CI, 9.1–33.3), and 27.8% (95% CI, 9.7–53.5), respectively. The highest risk was observed in patients with both DOR and age >37 years, with cancellation rates of 14.3% (95% CI, 5.4–28.5), 31.0% (95% CI, 15.3–50.8), and 50.0% (95% CI, 15.7–84.3) across the three exposure groups, yielding an overall cancellation rate of 24.1% (95% CI, 15.1–35.0). Conversely, women with normal ovarian reserve demonstrated substantially lower cancellation rates across all exposure durations (1.5% (95% CI, 0.6–3.1), 2.6% (95% CI, 1.2–4.9), and 6.3% (95% CI, 2.9–11.7)), as did women with PCOS (0.3% (95% CI, 0.0–1.5), 0.7% (95% CI, 0.1–2.4), and 1.0% (95% CI, 0.0–5.3)). The number needed to screen (NNS) to detect one progesterone-related cancellation for each risk group and estradiol exposure duration is provided in **Table 6**.

**Table 6:** Cycle cancellation rates by risk group and estradiol exposure duration.

Risk groups	Short (8–9 days) exposure	Intermediate (10–11 days) exposure	Prolonged (12–14 days) exposure	Overall
DOR	13/133 9.8% (5.3-16.1)	18/96 18.8% (11.5-28)	6/31 19.4% (7.5-37.5)	37/260 14.2% (10.2-19.1)
NNS	10 (6-19)	5 (4-9)	5 (3-13)	7 (5-10)
Age >37	6/77 7.8% (2.9-16.2)	9/47 19.1% (9.1-33.3)	5/18 27.8% (9.7-53.5)	20/142 14.1% (8.8-20.9)
NNS	13 (6-34)	5 (3-11)	4 (2-10)	7 (5-11)
DOR + Age >37	6/42 14.3% (5.4-28.5)	9/29 31.0% (15.3-50.8)	4/8 50.0% (15.7-84.3)	19/79 24.1% (15.1-35)
NNS	7 (4-18)	3 (2-7)	2 (1-6)	4 (3-7)
Normal reserve	7/468 1.5% (0.6-3.1)	9/346 2.6% (1.2-4.9)	9/142 6.3% (2.9-11.7)	25/956 2.6% (1.7-3.8)
NNS	67 (33-166)	38 (20-84)	16 (9-34)	38 (26-59)
PCOS	1/379 0.3.% (0.0-1.5)	2/304 0.7% (0.1-2.4)	1/102 1.0% (0.0-5.3)	4/785 0.5% (0.1-1.3)
NNS	379 (68-14970)	152 (42-1254)	102 (19-4029)	196 (77-719)

Values are presented as n/N, percentages (95% CI), or estimates (95% CI).

NNS, number needed to screen to detect one progesterone-related cancellation.

DOR, diminished ovarian reserve; PCOS, polycystic ovary syndrome.

## Discussion

To our knowledge, this is the first study to evaluate the impact of estradiol exposure duration on both progesterone-related cycle cancellation and reproductive outcomes within the same HRT–FET cohort. Our findings challenge the routine practice of empirically prolonging estradiol administration. In multivariable analysis, estradiol exposure beyond 11 days independently predicted cycle cancellation, with risk strongly influenced by patient characteristics—particularly advanced reproductive age and DOR. Importantly, extended estradiol exposure did not improve live birth outcomes, suggesting that prolonged administration may represent avoidable overtreatment in selected patients. Given the implications for healthcare costs, patient burden, and time to pregnancy, these results argue against a standardized HRT–FET protocol and support a more individualized approach to estradiol duration. Consistent with prior literature, younger age, double embryo transfer, and higher blastocyst quality independently predicted live birth. Overall, estradiol duration appears to be a modifiable determinant of cycle efficiency rather than merely a scheduling parameter.

Although hormonal monitoring in HRT cycles remains controversial, a uniform approach continues to be widely adopted in clinical practice. Standard HRT–FET protocols generally involve fixed estradiol exposure of 12–14 days or longer for logistical convenience, often without accounting for interindividual physiological variability or individualized cancellation risk, with reported progesterone elevation rates ranging from 1.9% to 7.4% (16–19). In contrast, our results indicate that avoiding estradiol exposure beyond 11 days is sufficient to maintain favorable reproductive outcomes while reducing progesterone-related cycle cancellation. Notably, some studies have reported comparable pregnancy rates with shorter (7-day) and longer (14-day) estradiol regimens; however, they did not evaluate cancellation rates (20, 21). Collectively, these findings suggest that progesterone elevation should not be considered inevitable but rather preventable to some extent and influenced by modifiable treatment factors, including estradiol duration and patient characteristics such as age and coexisting DOR or PCOS.

The increased cancellation risk observed with prolonged estradiol exposure is likely explained by interindividual variation in follicular phase length (22, 23). In women of advanced reproductive age or with DOR, the follicular phase tends to be shorter, increasing susceptibility to premature progesterone elevation when estrogen exposure exceeds physiological limits, particularly if hypothalamic–pituitary activity is not adequately suppressed. In our study, the overall cycle cancellation rate among women with DOR was 14.2% (NNS = 7), decreasing to 9.8% (NNS = 10) in the short exposure group, whereas the highest rate was observed in women with both DOR and age >37 years (24.1%, NNS = 4). In contrast, women with PCOS appeared relatively protected, likely due to reduced endogenous follicular activity, with an overall cancellation rate of 0.5% (NNS = 196). The NNS ranged from 379 in the short exposure group to 152 in the intermediate group among PCOS patients, underscoring the variability in monitoring yield across subgroups. Taken together, these findings suggest that standardized HRT–FET protocols assume biological uniformity within a heterogeneous population; estradiol duration interacts with ovarian reserve and age, and a uniform approach may increase avoidable cancellations without improving live birth outcomes.

Our findings also align with our previous study evaluating the omission of routine hormonal monitoring in HRT–FET cycles among good-prognosis women, which reported comparable pregnancy outcomes between monitored and unmonitored groups (24). In our cohort, progesterone-related cycle cancellation was uncommon in this population—particularly among women with PCOS—and remained low when estradiol exposure did not exceed 11 days. These findings suggest that, in carefully selected low-risk patients, routine progesterone monitoring may offer limited additional clinical benefit. Overall, the data support tailoring monitoring intensity and treatment strategies to individual patient risk profiles.

From a clinical perspective, our findings support a risk-stratified approach to HRT–FET management. Among good-prognosis patients—particularly those aged ≤37 years without DOR—limiting estradiol exposure to 10–11 days was associated with low cancellation rates without compromising reproductive outcomes. In this group, avoidable cancellations may be driven more by unnecessary prolongation of estrogen exposure than by intrinsic susceptibility to progesterone elevation. Conversely, in higher-risk patients, including those of advanced reproductive age or with DOR, alternative strategies—such as shorter estradiol exposure (8–9 days) or pituitary suppression-based or non-HRT–FET protocols—may better minimize delays and optimize time to live birth. Overall, these findings favor an individualized, physiology-tailored approach over uniform treatment protocols.

This study has several limitations. Its retrospective design introduces potential selection bias, unmeasured confounding, and limits causal inference. Although we adjusted for key confounders and included only first HRT–FET cycles to minimize within-patient bias, residual confounding cannot be excluded. Additionally, two different HRT–FET regimens were used during the study period. However, adjustment for treatment regimen in the multivariable analysis did not materially change the results, and the regimen itself was not independently associated with the primary outcome. Furthermore, in our routine clinical practice, estradiol duration was determined primarily by logistical considerations, including patient scheduling, travel arrangements, physician availability, weekends, and public holidays, rather than by endometrial development. Only a small proportion of patients initially assigned to the short or intermediate duration groups required prolonged estradiol administration to achieve an endometrial thickness of at least 7 mm. Patients assigned to prolonged estradiol exposure for logistical reasons were not routinely assessed before day 10. Therefore, we cannot determine whether some would have required prolonged treatment because of insufficient endometrial development. This limitation should be considered when interpreting the association between estradiol exposure duration and cycle cancellation due to progesterone elevation. Subgroup analyses, particularly among combined high-risk patients, should be interpreted cautiously due to smaller sample sizes. The findings from the smallest subgroup, particularly patients with DOR who were aged >37 years and had prolonged estradiol exposure, should be interpreted with caution because of the limited sample size. Likewise, the findings for the PCOS subgroup are presented as supportive rather than definitive, given the small number of events and the resulting wide confidence intervals. Despite these limitations, the study has notable strengths. All data points were manually double-checked to ensure data accuracy and completeness. To minimize variability related to repeated observations and treatment adaptation, only the first HRT–FET cycle per patient was included in the analysis, while subsequent cycles—including those following progesterone-related cancellation—were excluded. Moreover, all cycles were managed by a single senior physician (MRA), thereby reducing inter- and intra-observer variability in clinical decision-making and HRT–FET management.

In conclusion, progesterone elevation in HRT–FET cycles may be mitigated by optimizing estradiol exposure duration according to individual patient factors, such as ovarian reserve status and age, with direct implications for clinical practice: In good-prognosis patients (age ≤37 years, normal ovarian reserve, or PCOS), limiting estradiol exposure to 10–11 days appears sufficient to maintain low cancellation rates without compromising live birth outcomes. Routine progesterone monitoring may offer limited clinical benefit in PCOS patients, given the high number needed to screen (NNS = 196). Cancellation risk remains substantial in women with DOR (14.2%, NNS = 7), those aged >37 years (14.1%, NNS = 8), and particularly in women with both risk factors (24.1%, NNS = 4). In these patients, alternative strategies—such as shorter estradiol exposure (8–9 days), pituitary suppression-based, or non-HRT–FET protocols—may be more appropriate. Overall, these findings support a risk-stratified, individualized approach to HRT–FET management rather than uniform treatment protocols. Prospective studies are needed to validate these risk-stratified strategies.

## Data Availability

The raw data supporting the conclusions of this article will be made available by the authors, without undue reservation.
